# Screening of anti-inflammatory activities components of *Angelica dahurica* root based on spectrum-effect relationship analysis and NF-κB pathway

**DOI:** 10.3389/fphar.2024.1396001

**Published:** 2024-08-09

**Authors:** Huan Shi, Qianqian Wang, Yaqing Chang, Yuguang Zheng, Dan Zhang, Yunsheng Zhao, Long Guo

**Affiliations:** ^1^ Traditional Chinese Medicine Processing Technology Innovation Center of Hebei Province, Hebei University of Chinese Medicine, Shijiazhuang, China; ^2^ Hebei Chemical and Pharmaceutical College, Shijiazhuang, China

**Keywords:** *Angelica dahurica* root, anti-inflammatory, HPLC-Q/TOF-MS, spectrum-effect relationships, NF-κB

## Abstract

*Angelica dahurica* root (ADR), a commonly utilized herbal medicine in China and other Asian nations, which has anti-inflammatory effects on diverse inflammatory ailments. However, the bioactive components and underlying mechanism responsible for the anti-inflammatory effect of ADR are still unclear. This work attempted to discover the anti-inflammatory bioactive compounds and explore their underlying mechanism in ADR based on spectrum-effect relationship analysis and NF-κB signaling pathway. Chromatographic fingerprints of ADR samples were established by high performance liquid chromatography with diode array detection (HPLC-DAD), and a total of eleven common peaks were selected. Then, high performance liquid chromatography coupled with quadrupole time-of-flight mass spectrometry (HPLC-Q/TOF-MS) was employed for identification of eleven common peaks in ADR Meanwhile, the anti-inflammatory activities of ADR samples were assessed by inhibition of NO, interleukin-1β (IL-1β), interleukin-6 (IL-6) and tumor necrosis factor-α (TNF-α) production in LPS-induced RAW264.7 cells. The spectrum-effect relationships between the eleven common peaks in HPLC fingerprints and anti-inflammatory effects of ADR samples were investigated to identify the potential anti-inflammatory bioactive compounds by grey relational analysis (GRA) and partial least squares regression (PLSR). The spectrum-effect relationship analysis results indicated that six coumarin compounds, including bergapten, xanthotoxin, phellopterin, isoimperatorin, xanthotoxol and imperatorin could be potential anti-inflammatory bioactive compounds in ADR. The further validation experiments also showed that these six coumarins demonstrated significant inhibition of NO, IL-1β, IL-6, and TNF-α production in LPS-induced RAW264.7 cells. In addition, western blot analysis was conducted to explore the mechanisms of two potential anti-inflammatory bioactive compounds (phellopterin and isoimperatorin) by assessing the protein levels in the NF-κB signaling pathway. The western blot results illustrated that phellopterin and isoimperatorin could significantly down-regulate the phosphorylated NF-κB p65 (p-p65), phosphorylated IκBα (p-IκBα) and iNOS, and depress the pro-portion of p-p65/p65 and p-IκBα/IκBα, which indicated that these two coumarins in ADR could potentially exert anti-inflammatory effects by suppressing of NF-κB pathway.

## 1 Introduction

*Angelica dahurica* root (ADR), the dried roots of *A. dahurica* (Fisch. ex Hoffm.) Benth. et Hook.f. and *A. dahurica* (Fisch. ex Hoffm.) Benth. et Hook.f. var. formosana (Boiss.) Shan et Yuan, is a popular used herbal medicine to treat pain, cold fever, abscesses, rhinitis, toothache, furunculosis, acne and cold-damp pain in China, Korea and Japan (Zhao et al., 2022). Except as an important herbal medicine, ADR is also commonly used in foods as a spice to increase their fragrance and eliminate unpleasant odors. Previous researches demonstrated that ADR contains various bioactive constitutes, such as coumarins, essential oils, flavonoids and polysaccharides (Wang et al., 2023). Among the bioactive compounds, coumarins are the primary bioactive ingredients in ADR, which play a crucial role in herbal remedy (Shi et al., 2022). Multiple pharmacological studies have shown that coumarins contained in ADR has anti-inflammatory, anticancer, antidepressant, antiviral, vasodilation, antibacterial and antidiabetic effects (Lee et al., 2020; Luo et al., 2020; Banikazemi et al., 2021). Although the utilization of ADR as herbal medicine has a long history, there is still a limited amount of research on the phytochemicals and bioactive compounds. Several studies have demonstrated that some coumarins isolated from ADR had significant anti-inflammatory effects (Yang et al., 2015). However, the coumarins constituents responsible for the anti-inflammatory activities of ADR and their mechanism is still not fully clear (Kang SW et al., 2010). It is necessary to investigate and screen out main coumarins components that represent the anti-inflammatory properties of ADR.

It is commonly recognized that the therapeutic effects of herbal remedies are achieved through a comprehensive approach involving various components. Therefore, it is time-consuming and inefficient to screen bioactive compounds from herbal medicines by conventional methods of extraction, purification, structure identification and bioactive study of the compounds (Gong et al., 2020). The chemical fingerprint serves as a convenient and efficient method to assess the consistency and quality of herbal medicines, and also providing insights into their chemical characteristics to some extent (Chen et al., 2019). The spectrum-effect relationship analysis is a credible technique to associate the chemical fingerprints with biological activities of herbal medicines and to screen for bioactive components (Li et al., 2023). By merging pharmacodynamics research with chemical fingerprints data, the analysis of spectrum-effect relationships can effectively link the chemical components in fingerprints with their respective biological activities, and clarify the correlation between fingerprint characteristics and biological activity. Therefore, the spectrum–effect relationship analysis has been extensively utilized in the assessment and selection of active components from medicinal herbs.

Inflammation is a natural and protective immune response activated in response to external factors such as infections, injury, or chemical irritants. A properly functioning inflammatory response safeguards our body from internal injury and external invaders. However, dysregulation of inflammatory mediators may contribute to the progression of inflammatory disease pathogenesis (Fang et al., 2021). Therefore, controlling inflammation is a crucial focus for preventing and treating inflammatory disorders. Macrophages are the primary inflammatory and immune cells, which have a crucial function in the inflammatory process through the release of various pro-inflammatory mediators including interleukin-1β (IL-1β), interleukin-6 (IL-6), and tumor necrosis factor-α (TNF-α). Suppression of pro-inflammatory mediators and cytokines has been explored as a crucial strategy in the treatment of inflammatory disorders (Tian et al., 2021). Lipopolysaccharide (LPS) is derived from Gram-negative bacteria and serves as an endotoxin component. The RAW 264.7 macrophages stimulated by LPS are commonly utilized as a crucial cellular model for screening anti-inflammatory bioactive compounds and investigating the underlying mechanisms. Hence, this study evaluated the anti-inflammatory activities of ADR samples by inhibiting the production of NO, IL-1β, IL-6, and TNF-α in LPS-stimulated RAW264.7 macrophages. Furthermore, the spectrum-effect analysis methods, including grey relational analysis (GRA) and partial least squares regression (PLSR) were establish to explore the relationships between the phytochemical fingerprints and anti-inflammatory activities of ADR samples, and screen out the potential anti-inflammatory compounds in ADR. GRA is frequently utilized to effectively compare quantitative trends in dynamically changing systems (Li et al., 2024). PLSR is a powerful method of regression modeling handle multiple dependent variables in relation to multiple independent variables effectively, which can effectively decompose and filter the data to accurately predict outcomes (Chang et al., 2021).

Nuclear factor kappa-B (NF-κB) plays an important role in the occurrence and development of inflammatory diseases and is a classic and central transcription factor in the secretion of inflammatory mediators and cytokines in LPS-stimulated RAW 264.7 macrophages (Wang et al., 2022). In addition, NF-κB is composed of p50 and p65 subunits, which reside in the cytoplasm and interact with the IκB protein in inactive cells. Upon activation by LPS or other inflammatory triggers, IκB kinase rapidly phosphorylates and breaks down IκB within the IκB/NF-κB complex (Fang et al., 2021). Consequently, inhibition of the NF-κB signaling pathway is viewed as a pivotal target and an efficient treatment approach for inflammatory diseases. Meanwhile, NO is an inflammatory mediator produced by the catalytic action of the enzyme of inducible nitroxide synthase (iNOS) enzyme (Ke et al., 2021). Numerous researches have shown that an overabundance of iNOS could cause the excessive creation of the inflammatory factor NO. Based on the evidence presented, the protein expression of iNOS and NF-κB including IκBα, phosphorylated IκBα (p-IκBα), p65 and phosphorylated p65 (p-p65) pathways were evaluated using western blot analysis to investigate the underlying mechanism of potential anti-inflammatory compounds screened out in ADR.

In general, the primary objective of this study was to identify the anti-inflammatory bioactive components in ADR using a comprehensive approach. Firstly, HPLC-DAD method was developed to establish the chemical fingerprints of ADR samples. HPLC-Q/TOF-MS method was then utilized to specifically identify and characterize the coumarins present in the chemical fingerprints of ADR. The anti-inflammatory effects of different batches of ADR samples were assessed by measuring the suppression of NO, IL-1β, IL-6, and TNF-α production in LPS-induced RAW 264.7 macrophages. Furthermore, the spectrum-effect relationships between chemical fingerprints and anti-inflammatory activities of ADR samples were investigated by GRA and PLSR to determine the specific coumarins responsible for the observed anti-inflammatory activities. In addition, the potential anti-inflammatory coumarins screen out in ADR were further verified by the inhibitory effects on NO, IL-1β, IL-6, and TNF-α production in LPS-stimulated RAW 264.7 macrophages. The underlying mechanism of potential anti-inflammatory coumarins in ADR were also explored through NF-κB signaling pathways.

## 2 Materials and methods

### 2.1 Materials and reagents

A total of twenty batches of ADR samples were obtained from various regions in China. After fibrous roots removed, the ADR samples were dried in a constant temperature oven at 50°C. The sources of the samples detailed were shown in Supplementary Table S1. The voucher specimens, identified by Associate Professor Long Guo have been stored at Traditional Chinese Medicine Processing Technology Innovation Center of Hebei Province, Hebei University of Chinese Medicine.

Reference standards of xanthotoxol, xanthotoxin, bergapten, imperatorin and isoimperatorin and phellopterin were purchased from Chengdu Must Bio-Technoligy Co., Ltd. (Chengdu, China). Their purities were confirmed to be over 98% by HPLC-DAD analysis. Methanol, acetonitrile and formic acid of HPLC grade were obtained from Fisher Scientific (Pittsburgh, United States). Ultrapure water was generated using a Synergy water purification system (Millipore, United States). All other chemicals and reagents utilized were of analytical grade.

RAW264.7 mouse macrophages were purchased from the Cell Bank of the Chinese Academy of Sciences (Kunming, China). Fetal bovine serum and Dulbecco’s Modified Eagle’s Medium (DMEM) were purchased from GIBCO (New York, United States). Penicillin-streptomycin solution, pancreatic enzymes, LPS, cell counting kit-8 (CCK-8), IL-1β detection kit, IL-6 detection kit and TNF-α detection kit were purchased from Solarbio (Beijing, China). Sulfanilamide and N-(1-naphthyl) ethylenediamine dihydrochloride were purchased from Rhnwa (Shanghai, China). Dexamethasone (DEX) were purchased from Macklin Biochemcial (Shanghai, China). Sodium nitrite (NaNO_2_) was purchased from Tianjin Oubokai Chemical Co., Ltd. (Tianjin, China). Antibodies for β-actin, iNOS, p65, p-p65 (Ser536), IκBα, p-IκBα and secondary antibodies were purchased from Cell Signaling Technology (Boston, United States).

### 2.2 Sample preparation

The ADR samples were crushed into powders and sieved through a 0.30-mm mesh sieve. 0.5 g of the ADR powder was subjected to extraction in 15 mL of 80% (v/v) methanol using an ultrasonic extractor operating at 40 kHz and 300 W at room temperature for a duration of 50 min. Subsequent to the extraction, the mixed suspension was centrifuged at 13,000 rpm for 10 min, and the supernatant was then passed through a 0.22 μm membrane filter anterior to HPLC-DAD and HPLC-Q/TOF-MS analysis.

Standard solutions of xanthotoxol (0.85 mg/mL), xanthotoxin (1.20 mg/mL), bergapten (0.60 mg/mL), imperatorin (2.20 mg/mL), phellopterin (2.55 mg/mL) and isoimperatorin (2.60 mg/mL) were prepared by accurately weighing and dissolving in methanol. Then, the standard solutions were diluted with methanol to a series of accurate concentrations for the establishment of linearity. All the solutions were stored at 4°C until use.

For anti-inflammatory experiments, 0.5 g of the ADR powder was subjected to extraction in 15 mL of 80% (v/v) methanol using an ultrasonic extractor operating at 40 kHz and 300 W at room temperature for a duration of 50 min. Subsequent to the extraction, the mixed suspension was centrifuged at 13,000 rpm for 10 min, and 150 μL of the supernatant was dried in a Termovap Sample Concentrator (Hangzhou Miulab Instruments Co., Ltd.) at 30°C. Subsequently, the remaining residue was redissolved in 25 mL DMEM. Preceding the cell experiment, the solution was sieved through a 0.22 μm membrane. The concentrations of ADR sample solutions were determined based on the quantity of crude herbal medicine, resulting in a final concentration of 50 μg/mL.

### 2.3 HPLC-DAD and HPLC-Q/TOF-MS conditions

The HPLC-DAD analysis was carried on a Shimadzu LC-2030 HPLC system comprised a solvent delivery unit, autosampler, binary pump, column oven and photodiode array detector (Shimadzu, Kyoto, Japan). The column (Agilent ZORBAX SB C18, 4.6 mm × 250 mm, and 1.8 μm) was utilized for analysis. The mobile phases consisted of 0.1% formic acid in water (A) and acetonitrile (B). The gradient elution protocol as follows: 0–5 min, 10%–20% B; 5–15 min, 20%–35% B; 15–30 min, 35%–60% B; 30–35 min, 60%–70% B; and 35–36 min, 70%–100% B. The flow rate remained constant at 0.5 mL/min with the column temperature set at 25°C. Detection occurred at 250 nm wavelength, and a 5 μL sample injection volume was used. Data acquisition was carried out using Shimadzu Labsolutions.

The HPLC-Q/TOF-MS analysis was conducted using an Agilent 1290 HPLC system coupled with an Agilent 6545 quadrupole time-of-flight mass spectrometer system (Agilent Technologies, Santa Clara, CA, United States). Chromatographic separation was also performed on an Agilent ZORBAX SB C18 column (4.6 mm × 50 mm, 1.8 μm) and the HPLC chromatographic conditions were the same as HPLC-DAD analysis, the sample volume was set at 1 μL. The MS acquisition parameters were as follows: sheath gas temperature, 350°C; drying gas (N_2_) temperature, 320°C; drying gas (N_2_) flow rate, 10.0 L/min; sheath gas flow (N_2_) rate, 11 L/min; nebulizer gas pressure, 35 psi; capillary voltage, 3,500 V; fragmentor voltage, 135 V; collision energy, 40 eV. The analysis was performed in positive ion mode (ESI^+^) with the mass range of *m/z* 100–1,000 Da, and data acquisition was carried out using MassHunter Workstation.

### 2.4 HPLC fingerprints

#### 2.4.1 Method validation

The reliability of the HPLC-DAD method employed in the analysis of ADR samples was ensured by verifying its precision, repeatability, stability and linearity. The precision of the analysis was evaluated through injecting and analyzing the same sample for six consecutive times. Repeatability was assessed by the preparation and parallel analysis of six ADR samples. Meanwhile, the stability was determined by analyzing the same sample at 0, 4, 8, 12, 24 and 48 h at room temperature. The precision, stability, and repeatability of the established HPLC-DAD method were determined by calculating the relative standard deviations (RSDs) of peak areas corresponding to common peaks. Six level concentrations of working standard solutions were analysed to evaluate the linearity of the HPLC-DAD method. The calibration curves were calculated by plotting the peak areas of each analyte.

#### 2.4.2 Establishment and evaluation of HPLC fingerprints

The established HPLC-DAD method was used to analyze twenty batches of samples of ADR to gain chromatograms that included peak areas and retention times, and the data from the chromatograms was saved in CDF format. The HPLC fingerprints of the ADR samples were automatically matched by the Similarity Evaluation System for Chromatographic Fingerprint of Traditional Chinese Medicine (version 2012 A). The reference fingerprint chromatogram was created using the median method by comparing the chromatograms of the twenty batches of ADR samples. The software also calculated the similarity between the reference fingerprint chromatogram and the chromatographic profiles of the ADR samples.

### 2.5 Anti-inflammatory activities of ADR samples

#### 2.5.1 Determination of NO production

The concentration of nitrite in the medium was quantified to assess NO production through the Griess reaction. RAW264.7 macrophage cells were cultured in DMEM with 10% fetal bovine serum and 1% penicillin-streptomycin. The cells were incubated at 37°C in a humidified atmosphere containing 5% CO_2_. Briefly, cells were seeded in a 96-well plate at a density of 5 × 10^5^ cells per well. Following a 24-h incubation period, the cells were treated with ADR extract for 1 h, then stimulated with LPS (1 μg/mL) for 24 h. Subsequently, 100 µL of culture supernatant was combined with an equal volume of Griess reagent (1% sulfanilamide in water and 0.1% naphthylethylenedi-amine dihydrochloride in 5% phosphoric acid) and allowed to react at room temperature in the dark for 15 min before measuring absorbance at a wavelength of 540 nm with a microplate reader. Standard curve was generated using NaNO_2_, and nitrite concentrations in the media were determined. DEX (10 μg/mL) was utilized as a positive control. The inhibition rate of NO production was calculated using a specific formula:

$$\text{Inhibition }\text{ratio }\text{of }\text{NO }\text{production }\left(\%\right)=1-\frac{\text{Cs}-\text{Cn}}{\text{Cm}-\text{Cn}}\times 100$$

C_m_ is the NO concentration of model group, C_s_ is the NO concentration of sample group and C_n_ is the NO concentration of normal control group.

The viability of cells was assessed through the CCK-8 assay. Cells were plated on 96-well plates at a density of 2 × 10^5^ cells/well, and cultured in serum-deprived medium with the concentrations of ADR samples (100 μg/mL) for 24 h. Following a 3 h incubation period, the CCK-8 reagent was introduced, and the absorbance (OD) was quantified at 450 nm using a microplate reader. CCK-8 reagent was then added and the absorbance (OD) at 450 nm was measured using a microplate reader. Cell viability was determined by comparing the results to those of the control group. All experiments were performed in triplicate.

#### 2.5.2 Determination of IL-1β, IL-6, and TNF-α production

After the incubation of cells with LPS, DEX, or ADR extract for 24 h, the RAW 264.7 cell culture supernatants were collected and processed. The supernatant was obtained by centrifuging the medium at 1,000 rpm at 4°C for 10 min. Cell culture supernatants were added to ELISA plates to measure the levels of IL-1β, IL-6, and TNF-α as the respective instructions. Each sample was tested in triplicate.

### 2.6 Spectrum-effect relationship analysis

#### 2.6.1 Gray relational analysis

GRA could express the interconnections among different factors, which is commonly used in spectrum-effect relationship analysis of herbal medicines (Chen et al., 2021; Wu et al., 2024). The areas of eleven common peaks in HPLC fingerprints of ADR samples were considered as the sub-sequence, and the anti-inflammatory effects were considered as the parent sub-sequence. Then, the gray correlation degree (GRD) was calculated to determine the contribution of these common peaks on the anti-inflammatory effects of ADR samples with a distinguishing coefficient set as 0.8. The higher the value of GRD was, the stronger activities of the common peaks could be.

#### 2.6.2 Partial least squares regression analysis

PLSR is a regression model in statistics that can effectively address the issue of multicollinearity between a group of independent variables and a set of dependent variables (Jiang et al., 2024). In this study, PLSR was utilized to analyze the relationship between the common peaks and anti-inflammatory effects of ADR samples. The eleven common peak areas were considered as the independent variable (X), while the anti-inflammatory activities were determined by assessing the levels of NO, IL-1β, IL-6, and TNF-α was considered as the dependent variable (Y). The PLSR model was established using Simca-P14.1 (Umetrics, Umea, Sweden) software and the regression coefficient was utilized to demonstrate the impact of the independent variables on the dependent variables.

### 2.7 Verification of the anti-inflammatory activity

#### 2.7.1 Determination of NO, IL-1β, IL-6, and TNF-α production

To further verify the anti-inflammatory activities of the potential anti-inflammatory compounds in LPS-induced macrophages, secretion of NO and IL-1β, IL-6, and TNF-α were assessed by Griess reaction and ELISA kits, respectively.

#### 2.7.2 Western blot analysis

The western blot assay was utilized to determine protein expression. Briefly, RAW 264.7 macrophages were first seeded culturing for 24 h and pretreated with different concentrations of potential anti-inflammatory compounds for 1 h, then LPS (1 μg/mL) was added for 24 h of treatment. Subsequently, the collected cells were lysed in RIPA lysis buffer to extract the total protein, and the quantification of the protein concentration was measured using the BCA protein kit. The concentration of the protein was measured using the BCA protein kit. Following electrophoresis of protein on 10% SDS-PAGE gels and transfer onto PVDF membranes. The membranes were blocking for 3 h at room temperature with 5% nonfat dry milk and then incubated overnight at 4°C with the following primary antibodies: β-actin (1:10,000), iNOS (1:1,000), p65 (1:1,000), p-p65 (Ser536) (1:1,000), IκBα (1:1,500) and p-IκBα (1:1,500). After washing the membranes thrice for 10 min with 1 × TBST, the secondary antibodies were (1:20,000) incubated for 1 h. Finally, chemiluminescence was used to visualize the immunoreactive bands, which were quantified using Alpha Analysis Software through densitometry. Data were normalized to the level of β-action.

## 3 Results and discussions

### 3.1 HPLC fingerprints

#### 3.1.1 Optimization of HPLC-DAD condition

To efficiently separate ADR samples using HPLC-DAD, various conditions such as mobile phases, column temperatures, flow rates, and detective wavelengths were compared and optimized. Among the different mobile phases tested, formic acid water-acetonitrile with gradient elution proved to be the most effective for separating the analytes. The column temperature was maintained at 25°C, and a flow rate of 0.50 mL/min was chosen for optimal results. By setting the detective wavelength at 310 nm, the majority of common peaks showed stable and strong absorption intensity, leading to the best response signal for the determined compounds. These optimized HPLC-DAD conditions led to rapid and efficient separation of the ADR samples.

#### 3.1.2 Method validation

The precision, repeatability and stability of the established HPLC-DAD method were validated. The results in Supplementary Table S2 demonstrated that the precision presented as RSDs of peak areas for the eleven common peaks were less than 1.29%. The repeatability presented as RSDs was less 1.97%, and the stability was less than 1.72%. As shown in Supplementary Table S3, the correlation coefficient values (R^2^ ≥ 0.9990) showed satisfactory linearity between the concentration and peak area of six analytes within the linearity range. The method validation results clearly indicate that the established HPLC-DAD method is reliable and appropriate for conducting fingerprint analysis of ADR samples.

#### 3.1.3 HPLC fingerprints establishment and similarity analysis

**FIGURE 1:**
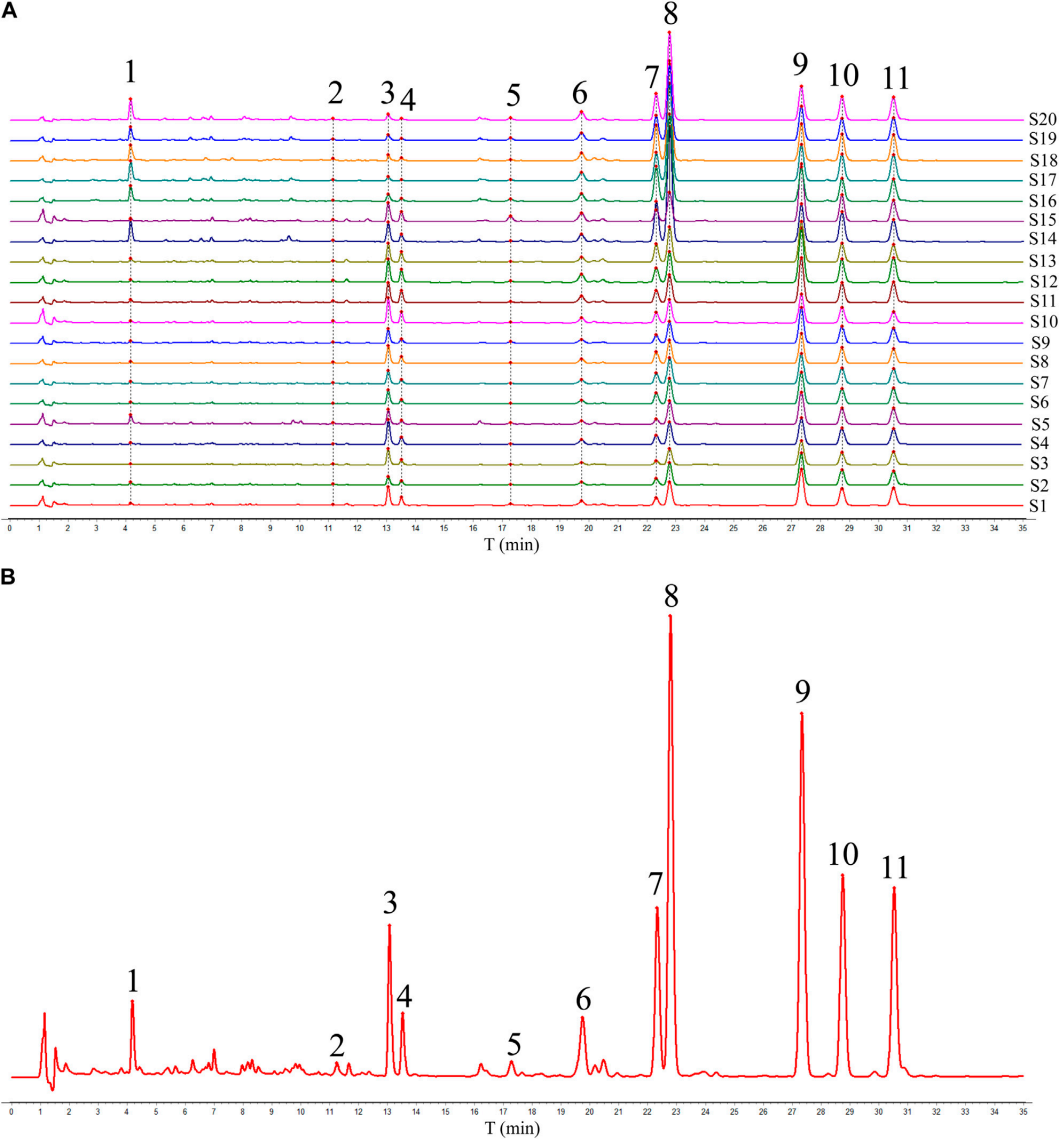
HPLC fingerprints **(A)** and reference fingerprint **(B)** of twenty batches of ADR samples.

Twenty batches of ADR samples obtained from diverse regions were subjected to the validated HPLC-DAD analysis under optimized condition, and the chromatogram of ADR samples were shown in Figure 1A. Subsequently, the HPLC fingerprints were constructed based on the chromatograms of ADR samples, and the median method were used to generate reference fingerprints after multi-point calibration and data alignment using the similarity evaluation software (Similarity Evaluation System for Chromatographic Fingerprints of Traditional Chinese Medicines). As shown in Figure 1B, eleven distinctive peaks present in all the ADR samples with clear segregation and resolution were identified as the common peaks, which illustrated the similarity between the different samples.

The comparison of the HPLC chromatograms of ADR samples with the reference fingerprint was conducted, and similarity values were determined using the correlative coefficient and cosine value of vectorial angle through the evaluation software for similarity. As shown in Table 1, the similarity values ranged from 0.930 to 0.984 between HPLC fingerprint of each ADR sample and the reference fingerprint. The analysis of similarity results suggested that there were similar chemical compositions among different batches of ADR samples.

**TABLE 1:** Similarity of 20 batches of ADR samples.

Sample number	Similarity	Sample number	Similarity
S1	0.956	S11	0.952
S2	0.963	S12	0.951
S3	0.940	S13	0.983
S4	0.956	S14	0.939
S5	0.959	S15	0.930
S6	0.979	S16	0.952
S7	0.984	S17	0.952
S8	0.970	S18	0.950
S9	0.949	S19	0.970
S10	0.955	S20	0.955

### 3.2 Characterization of common peaks in ADR by HPLC-Q/TOF-MS

**FIGURE 2:**
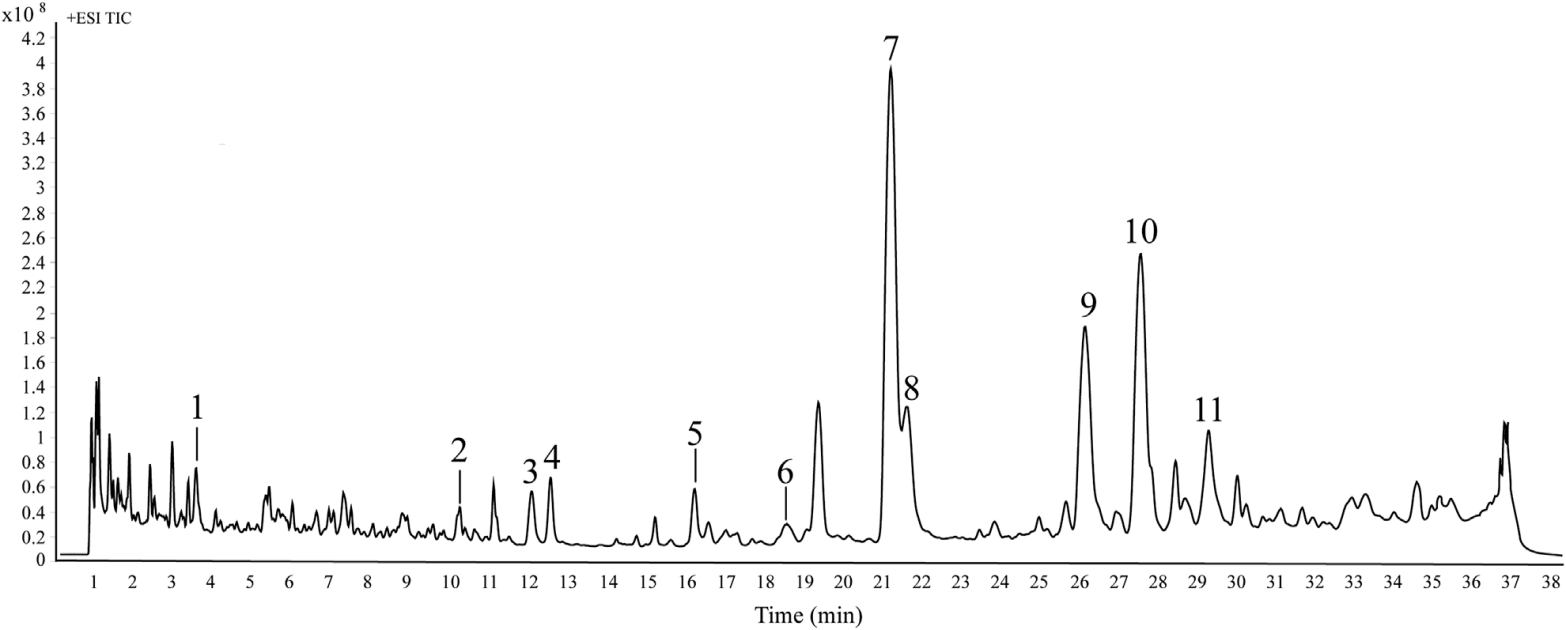
The typical total ion chromatograms of ADR samples in positive ion mode by HPLC-Q/TOF-MS. The peak numbers are consistent with the compound numbers presented in Table 2.

According to the results of HPLC fingerprint, eleven peaks (1–11) were selected as the common peaks of ADR samples. An HPLC-Q/TOF-MS method was also utilized to quickly identify the eleven common peaks in HPLC fingerprints. The total ion chromatogram (TIC) of ADR sample in positive ion mode is shown in Figure 2. Based on previous literature, fragmentation behaviors, retention time, major fragment ions, retention time and reference standards, a total of eleven coumarins were identified, including scopolin, xanthotoxol, oxypeucedanin hydrate, byakangelicin, xanthotoxin, bergapten, byakangelicol, oxypeucedanin, imperatorin, phellopterin, and isoimperatorin (Zheng et al., 2010; Li et al., 2014). The information of identification, including retention time, chemical formula, and fragment ions was shown in Table 2. The chemical structures of the recognized elven coumarins are displayed in Supplementary Figure S1, and the MS and MS/MS spectrums of the eleven coumarin compounds was shown in Supplementary Figure S2.

**TABLE 2:** HPLC-Q/TOF-MS information for identification of coumarin compounds in ADR samples.

No	T_R_ (min)	Formula	Theoretical mass (*m/z*)	Measured mass [M + H]^+^	(+) ESI-MS/MS (*m/z*)	Error (ppm)	Identification	Reference
1	3.42	C_16_H_18_O_9_	354.0951	355.1039	193.0498 (100), 178.0261 (42.62), 163.0392 (50.64), 133.0282 (67.98), 117.0333 (31.67)	1.69	Scopolin	Zheng et al. (2010)
2	10.22	C_11_H_6_O_4_	202.0266	203.0342	175.0391 (31.15), 147.0443 (100), 131.0489 (28.32)	0.54	Xanthotoxol	Zheng et al. (2010)
3	12.05	C_16_H_16_O_6_	304.0947	305.1041	203.0346 (50.17), 175.0392 (4.95), 159.0443 (16.21), 147.0446 (100), 131.0494 (30.23), 119.0492 (8.94)	−0.93	Oxypeucedanin hydrate	Li et al. (2014)
4	12.53	C_17_H_18_O_7_	334.1053	335.114	233.0451 (36.82), 218.0215 (94.03), 203.0342 (6.14), 190.0265 (14.86), 173.0235 (100), 162.0308 (24.41)	1.56	Byakangelicin	Li et al. (2014)
5	16.22	C_12_H_8_O_4_	216.0423	217.0503	202.0263 (100), 185.0234 (11.74), 174.0311 (30.29), 161.0597 (35.57), 146.0363 (3.87)	0.77	Xanthotoxin^a^	^a^
6	18.60	C_12_H_8_O_4_	216.0423	217.0503	202.0261 (27.89), 174.0312 (85.95), 146.0362 (45.48), 131.0490 (9.61), 118.0415 (100), 90.0465 (95.98)	0.74	Bergapten^a^	^a^
7	21.19	C_17_H_16_O_6_	316.0947	317.1035	231.0290 (11.64), 218.0217 (84.84), 188.0110 (55.16), 175.0394 (100), 160.0158 (29.25), 145.0284 (14.44)	1.68	Byakangelicol	Li et al. (2014)
8	21.67	C_16_H_14_O_5_	286.0841	287.0922	203.0342 (13.5), 175.0390 (1.84), 159.0439 (6.22), 147.0445 (46.69), 59.0496 (100)	1.40	Oxypeucedanin	Zheng et al. (2010)
9	26.17	C_16_H_14_O_4_	270.0892	271.0975	203.0342 (100), 185.0236 (3.14), 175.0390 (8.99), 147.0442 (13.55), 131.0489 (3.64)	1.14	Imperatorin^a^	^a^
10	27.52	C_17_H_16_O_5_	300.0998	301.1083	218.0217 (100), 202.0261 (6.73), 190.0263 (11.19), 173.0233 (13.96), 162.0312 (27.5), 134.0363 (25.28)	1.78	Phellopterin^a^	^a^
11	29.34	C_16_H_14_O_4_	270.0892	271.0975	203.0344 (100), 175.0390 (2.04), 159.0440 (5.07), 147.0444 (9.99), 131.0490 (3.34)	1.44	Isoimperatorin^a^	^a^

^a^ Compounds identified with reference standards.

### 3.3 Anti-inflammatory activities of ADR samples

#### 3.3.1 Inhibition of NO production

To assess the cytotoxic impact of ADR extract on RAW 264.7 cells, the viability cells was firstly evaluated by the CCK-8 assay. RAW 264.7 macrophages were processed with different concentrations (0, 12.5, 25, 50, 100, and 200 μg/mL) of ADR extract for 24 h. The CCK-8 assay results (Supplementary Figure S3) indicated that there was no notable variance in viability levels between the control group and the group treated with all concentrations of ADR extract. The results suggests that the ADR extract did not cytotoxic effects on RAW264.7 cells within the specified test concentrations ranges, which were equivalent to 0–200 μg/mL of the raw herbal medicine.

**FIGURE 3:**
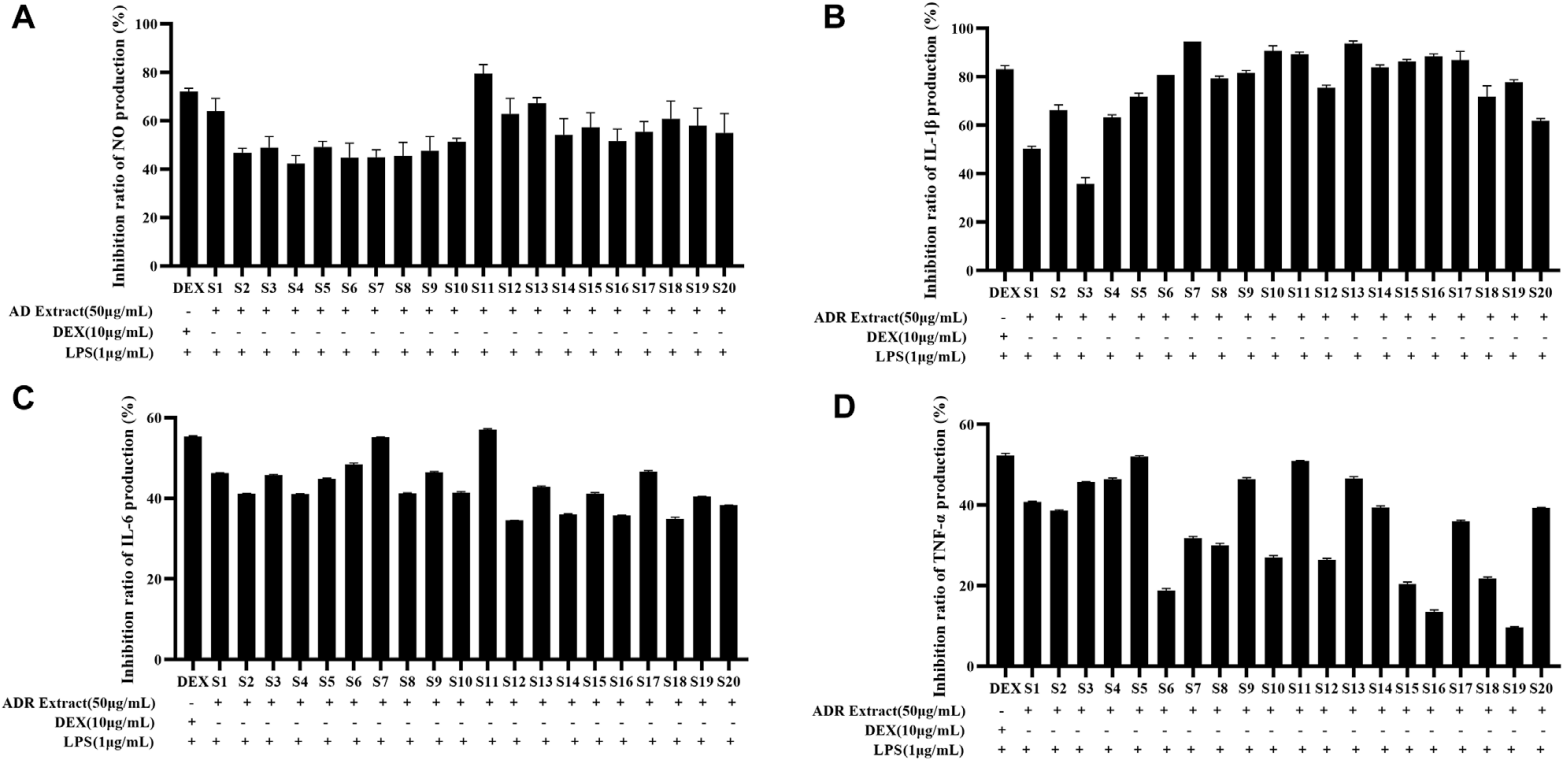
Inhibition ratio of NO **(A)**, IL-1β **(B)**, IL-6 **(C)**, and TNF-α **(D)** production in LPS-stimulated RAW264.7 cells treated with ADR samples extract (50 μg/mL).

The depression of NO production serves as a direct indicator of the anti-inflammatory effect due to close relationship between excessive NO production and the triggering of pro-inflammatory cytokines. The current investigation first evaluated the anti-inflammatory activities of ADR samples by determining the inhibition rate of NO production in LPS-induced RAW264.7 cells using the Griess assay reaction. The effects of ADR samples extract (50 μg/mL) on NO production in LPS-stimulated RAW264.7 cells were shown in Supplementary Figure S4. As presented in Figure 3A, all the twenty batches of ADR samples exhibited considerable inhibition of NO production with the rate of 42.39%–79.54% at the concentration 50 μg/mL raw herbal medicine. It could be noted that different batches of ADR samples showed significantly differences in the inhibition of NO production, potentially resulting from differences in bioactive constituents present in ADR samples. Previous studies reported that ADR samples extracted with different solvents (70% ethanol, ethanol, 70% methanol, methanol, and water) at 400 μg/mL concentration could significantly suppressed NO production in LPS-stimulated RAW 264.7 cells, but the inhibitory effects were inconsistent with the present experiment (Wang et al., 2016). The reason for the difference might be due to the different extraction solvents for the ADR samples.

#### 3.3.2 Inhibition of pro-inflammatory cytokines production

The potential anti-inflammatory activities of ADR samples were assessed by studying their inhibition on the release of pro-inflammatory cytokines such as TNF-α, IL-1β, and IL-6. The RAW264.7 cells were treated with LPS in presence or absence of ADR extract (50 μg/mL raw herbal medicine), and the concentrations of IL-1β, IL-6, and TNF-α were assessed using ELISA kits. The effects of ADR samples extract (50 μg/mL) on IL-1β, IL-6, and TNF-α production in LPS-stimulated RAW264.7 cells were presented in Supplementary Figure S4. As shown in Figures 3B–D, the ADR extract treatment significantly suppressed the productions of IL-1β, IL-6, and TNF-α in LPS-stimulated RAW264.7 cells (*p* < 0.05), which indicated that ADR might exhibit anti-inflammatory activities by reducing the release of pro-inflammatory cytokines releases.

To sum up, the results of the anti-inflammatory experiments indicated a significant reduction in NO, IL-1β, IL-6, and TNF-α levels in LPS-induced RAW264.7 cells treated with ADR samples. It was clear that the variability of anti-inflammatory among different batches of ADR samples was evident, which demonstrated that differences in the bioactive ingredient content across different batches of ADR samples to a certain extent. Variations in the anti-inflammatory effects might be attributed to the variation of bioactive components present in different samples of ADR samples. It is necessary to investigate the latent relevance between the bioactive compounds and anti-inflammatory effects, and screen out the potential anti-inflammatory constituents in ADR samples based on spectrum-effect relationship analysis.

### 3.4 Spectrum-effect relationship analysis

#### 3.4.1 Grey relational analysis

GRA is a versatile and efficient multi-variable statistical method capable of revealing correlations between multiple objects with limited information, which is widely used to solve problems involving multiple factors and complex relationships between variables (Chang et al., 2022).

**FIGURE 4:**
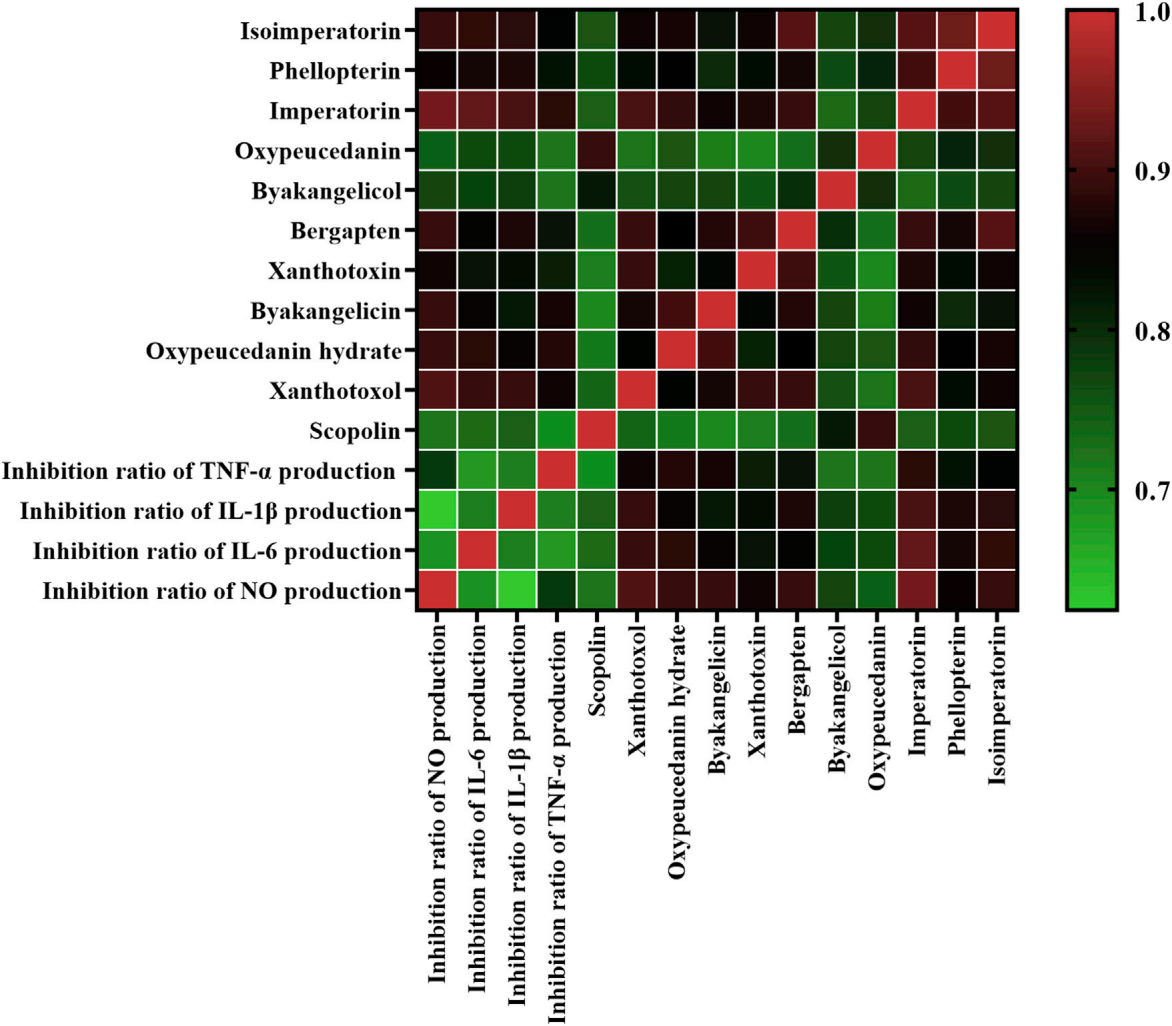
Heatmap analysis of grey relational analysis of eleven common peak areas and anti-inflammation activities. Red represents high correlated and green indicates low correlated.

In this present work, GRA was conducted to evaluate the connection between the peak areas of eleven common peaks (Supplementary Table S4) in HPLC fingerprints and anti-inflammatory activities of ADR samples. We obtained the information sequences of the peak areas of eleven common peaks in HPLC fingerprints of different batches of ADR samples as subsequences, and the four inflammatory indicators (inhibition rates of NO, IL-1β, IL-6, and TNF-α) of different batches of ADR samples as parent sub-sequence. The GRD was used to explain the relationship between common peaks and anti-inflammatory effects, and the GRD of the eleven common peaks in ADR fingerprints were calculated. The results (Figure 4) showed that the GRD of eight common peaks including xanthotoxol, oxypeucedanin hydrate, byakangelicin, xanthotoxin, bergapten, imperatorin, phellopterin, and isoimperatorin were more than 0.8, which demonstrated that these eight coumarin compounds contributed strongly to the anti-inflammatory effects of ADR. Furthermore, the higher the values of GRD were, the stronger anti-inflammation activities the coumarin compounds had.

#### 3.4.2 Partial least squares regression analysis

**FIGURE 5:**
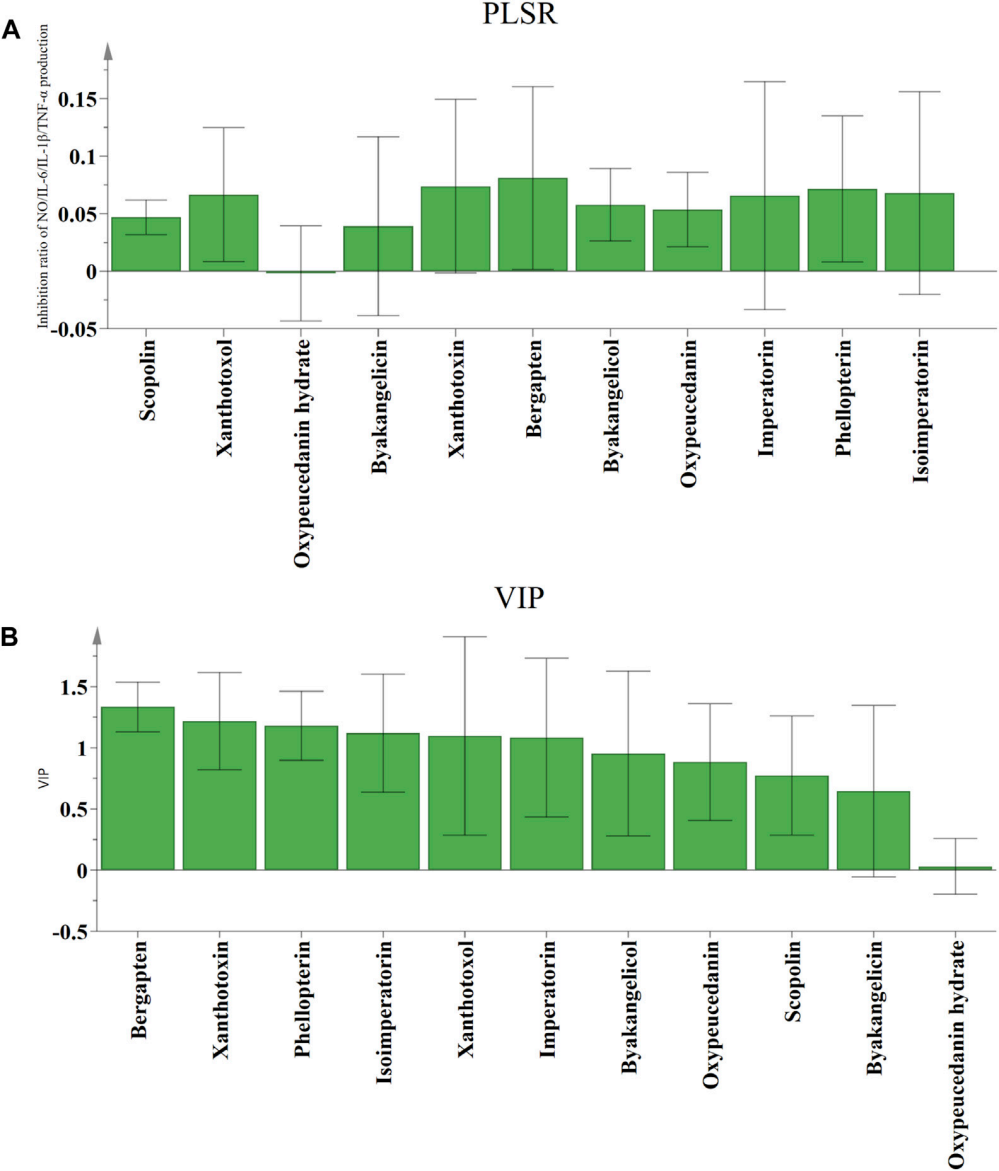
The correlation analysis by PLSR. Regression coefficients **(A)** and VIP values **(B)** between eleven common peaks and anti-inflammation activities.

PLSR is a powerful regression modeling method for exploring and understanding the intricate connections among various factors in a systematic and comprehensive manner, which addresses the issue of multiple dependent variables and multiple independent variables through the integration of multiple linear regression, canonical correlation analysis, and principal component analysis (Aghdamifar et al., 2023). The independent variable X was defined as the peak areas of the eleven common peaks, while the dependent variable Y was the anti-inflammatory activities (inhibition rates of NO, IL-1β, IL-6, and TNF-α). PLSR models were established, the regression coefficients and the variable importance in projection (VIP) values were determined. As shown in Figure 5, scopolin, xanthotoxol, byakangelicin, xanthotoxin, bergapten, byakangelicol, oxypeucedanin, imperatorin, phellopterin, and isoimperatorin were positively related to anti-inflammatory activities of ADR samples. As the regression coefficients values increased, the coumarin compounds exhibited stronger anti-inflammatory effects. The ranking of the regression coefficients for the coumarin compounds was as follows: bergapten > xanthotoxin > phellopterin > isoimperatorin > xanthotoxol > imperatorin > byakangelicin > oxypeucedanin > scopolin > byakangelicin > oxypeucedanin hydrate.

The VIP values serve as indicators of the significance of the variables, and peaks that correspond to variables with VIP values exceeding 1.0 could be considered to be responsible for anti-inflammatory activities of ADR samples. Six coumarin compounds, including bergapten, xanthotoxin, phellopterin, isoimperatorin, xanthotoxol, and imperatorin were selected with the VIP values greater than 1.0.

According to the results of GRA and PLSR analysis, some certain constituents contained in ADR samples had significant contribution to the anti-inflammatory activities. The spectrum-effect relationship analysis results indicated that bergapten, xanthotoxin, phellopterin, isoimperatorin, xanthotoxol, and imperatorin were considered as potential anti-inflammatory ingredients of ADR.

### 3.5 Verification of the anti-inflammatory activity

#### 3.5.1 Inhibition of NO, IL-1β, IL-6, and TNF-α production

**FIGURE 6:**
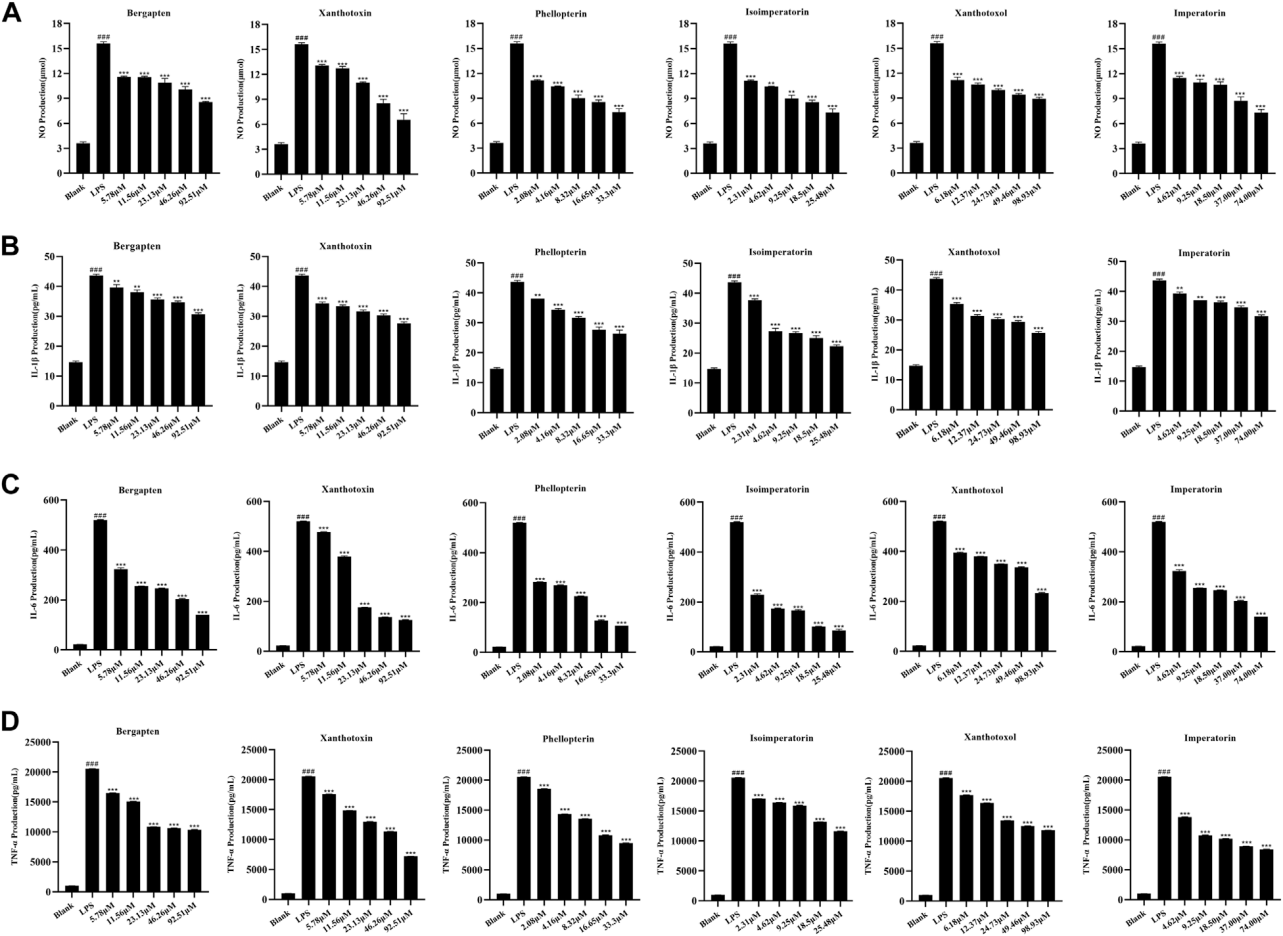
Effects of bergapten, xanthotoxin, phellopterin, isoimperatorin, xanthotoxol, and imperatorin on NO **(A)**, IL-1β **(B)**, IL-6 **(C)**, and TNF-α **(D)** production in LPS-stimulated RAW264.7 cells.^###^ *p* < 0.001, ***p < 0.001.

The above spectrum-effect relationship analysis illustrated that bergapten, xanthotoxin, phellopterin, isoimperatorin, xanthotoxol, and imperatorin could be the potential anti-inflammatory compounds in ADR. In order to confirm the accuracy of the results, the effects of the six coumarins on reducing inflammation were tested by assessing their ability to inhibit the production of NO, IL-1β, IL-6, and TNF-α in LPS-induced RAW264.7 macrophage cells. The CCK-8 assay results (Supplementary Table S5) indicated that there was no significant difference in cell viability between the control group and the group treated with six coumarins at different concentrations. Then, the inhibition of the six selected coumarins on NO, IL-1β, IL-6, and TNF-α production in LPS-induced RAW264.7 macrophage cells were investigated. The results (Figure 6) showed that all the six coumarins certain and dose-dependent inhibitory effects on NO, IL-1β, IL-6, and TNF-α production. The IC50 values of bergapten, xanthotoxin, phellopterin, isoimperatorin, xanthotoxol, and imperatorin for the release of NO, IL-1β, IL-6, and TNF-α in LPS-induced RAW264.7 macrophage cells were also calculated. As shown in Table 3, phellopterin and isoimperatorin exhibited the relatively lower IC50 values on NO, IL-1β, IL-6, and TNF-α production among the six potential anti-inflammatory constituents in ADR, which indicated that phellopterin and isoimperatorin have relatively strong anti-inflammatory effects. Previous investigation has reported that isoimperatorin showed anti-inflammatory activity by decreasing the production of TNF-α, IL-6, and IL-1β in LPS-activated RAW264.7 cells, which was in basic consistence with the results of this study (Wu et al., 2024). Meanwhile, there is evidence that phellopterin displayed inhibitory activity against NO production in LPS-activated RAW 264.7 macrophage cells (Deng et al., 2015). Therefore, phellopterin and isoimperatorin were selected for further verification of underlying mechanism of the potential anti-inflammatory compounds in ADR.

**TABLE 3:** The inhibitory effects of six coumarins on NO, IL-1β, IL-6, and TNF-α production in LPS-induced RAW264.7 cells (IC50 values, μM).

	Bergapten	Xanthotoxin	Phellopterin	Isoimperatorin	Xanthotoxol	Imperatorin
NO	53.79	41.51	8.03	4.31	32.69	21.90
IL-1β	149.00	37.21	15.60	6.48	60.92	175.50
IL-6	12.29	91.79	3.20	1.38	18.99	3.46
TNF-α	51.50	114.20	19.09	40.65	42.10	15.06

#### 3.5.2 Suppression on LPS-stimulated iNOS and NF-κB activation in RAW 264.7 macrophages

**FIGURE 7:**
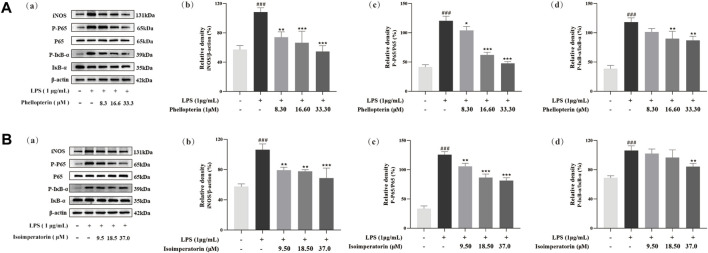
Effect of phellopterin **(A)** and isoimperatorin **(B)** on iNOS and NF-κB signaling pathway in LPS-induced RAW 264.7 macrophages. (a) Pictures of the protein levels of iNOS, p65, p-p65, IκBα, and p-IκBα analyzed by Western blot. (b) Relative density of iNOS. (c) Statistical analysis for ratios of p-p65/p65. (d) Statistical analysis for ratios of p-IκBα/IκBα. Each bar represents the mean ± SD (n = 3). ^###^ *p* < 0.001 compared to the un-stimulated control cells; *p < 0.05 compared to the LPS-stimulated cells; **p < 0.01 compared to the LPS-stimulated cells; ***p < 0.001 compared to the LPS-stimulated cells.

In the current research, we have selected the potential anti-inflammatory components in ADR, but the underlying mechanism remained uncertain. In general, iNOS play a vital role in the inflammatory process through the production of NO. Based on the significant suppression of NO by phellopterin and isoimperatorin, western blot analysis was used to measure the protein levels of iNOS. As shown in Figure 7, iNOS protein expression was remarkably up-regulated with LPS treatment, whereas the ratios decreased in phellopterin and isoimperatorin treatment groups dose-dependently manner in LPS-induced RAW 264.7 macrophages.

NF-κB is a key factor for the transcription of various inflammatory mediators, chemokines and other genes in the inflammatory response. When NF-κB is activated, it moves into the nucleus to initiate the transcription of inflammatory factors and further enhancing the production of pro-inflammatory cytokines. To further explore the underlying anti-inflammatory mechanism of the potential anti-inflammatory components phellopterin and isoimperatorin in ADR, western blot assays were used to analyze the protein expression of the NF-κB signaling pathway. As shown in Figure 7, western blot analysis demonstrated that the levels of p-p65 and p-IκBα protein induced by LPS was significantly higher than that of the control group. Phellopterin and isoimperatorin did not impact total p65 and IκBα protein levels, but they effectively inhibited the expression of both p-p65 and p-IκBα. Moreover, the ratio of p-p65/p65 and p-IκBα/IκBα was markedly down-regulated by phellopterin and isoimperatorin dose-dependently. Phellopterin and isoimperatorin demonstrated the ability to block NF-κB activation NF-κB activation by inhibiting phosphorylation levels of p65 and IκBα, thereby inhibiting the production of pro-inflammatory cytokines, thus achieving the purpose of treatment and prevention of inflammatory diseases.

## 4 Conclusion

This study looked at screening of the anti-inflammatory bioactive compounds in ADR and investigating their underlying anti-inflammatory mechanism. Firstly, the HPLC fingerprints were established based on the chromatograms of different batches of ADR samples, and eleven commons peaks were selected. Then, the eleven common peaks in HPLC fingerprints were characterized by HPLC-Q/TOF-MS. The anti-inflammatory activities of different batches of ADR samples were assessed by inhibiting the production of NO, IL-1β, IL-6, and TNF-α in LPS-induced RAW264.7 macrophage cells. The connections between chemical constituents and anti-inflammatory effects were investigated by spectrum-effect relationship analysis, and the results illustrated that bergapten, xanthotoxin, phellopterin, isoimperatorin, xanthotoxol, and imperatorin were potential anti-inflammatory constituents in ADR.

The anti-inflammatory activities of the six coumarins were validated by repression of NO, IL-1β, IL-6 and TNF-α production, and phellopterin and isoimperatorin exhibited the most significant anti-inflammatory activities among the six coumarin components. Moreover, the western blot results demonstrated that phellopterin and isoimperatorin could diminish the expression of iNOS and the phosphorylation of p65 and IκBα, which indicated that these two coumarins in ADR might exert anti-inflammatory by inhibiting of iNOS and NF-κB. The current study developed a comprehensive method utilizing HPLC fingerprints, HPLC-Q/TOF-MS and spectrum-effect relationship analysis to investigate the bioactive ingredients in herbal medicines, and also provided experimental data about the bioactive compositions and underlying mechanism for the anti-inflammatory activity of ADR.

## Data Availability

The original contributions presented in the study are included in the article/Supplementary Material, further inquiries can be directed to the corresponding authors.
