# Supplementary material for: Screening of anti-inflammatory activities components of Angelica dahurica root based on spectrum-effect relationship analysis and NF-κB pathway
Source: Front Pharmacol. 2024 Aug 9;15:1396001. doi: 10.3389/fphar.2024.1396001 (PMC11341442; doi:10.3389/fphar.2024.1396001)
Supplement: Supplementary file 1 [file DataSheet1.docx]

Supplementary Material





**Supplementary Figure 1** Chemical structures of eleven coumarins identified in ADR.


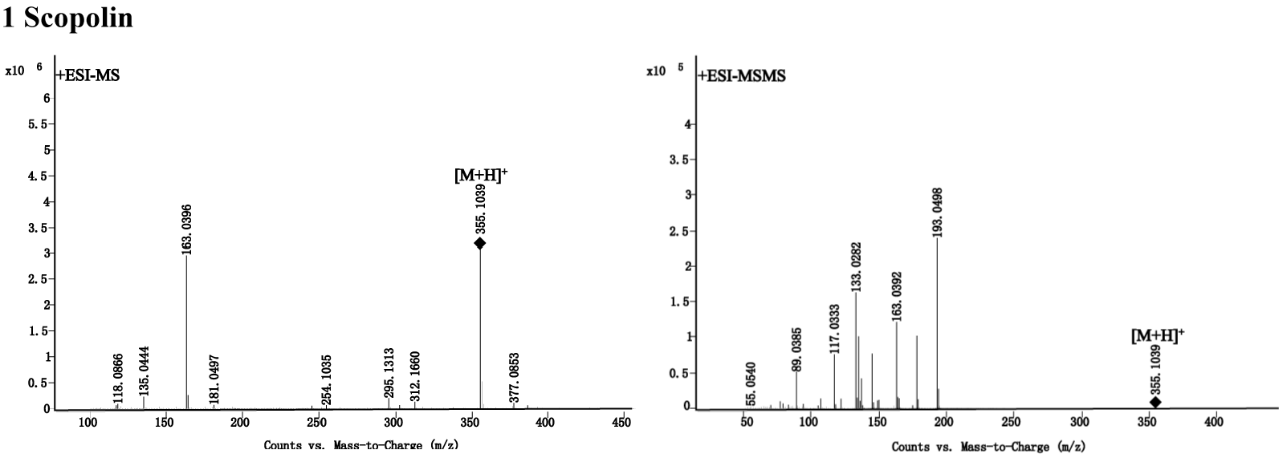


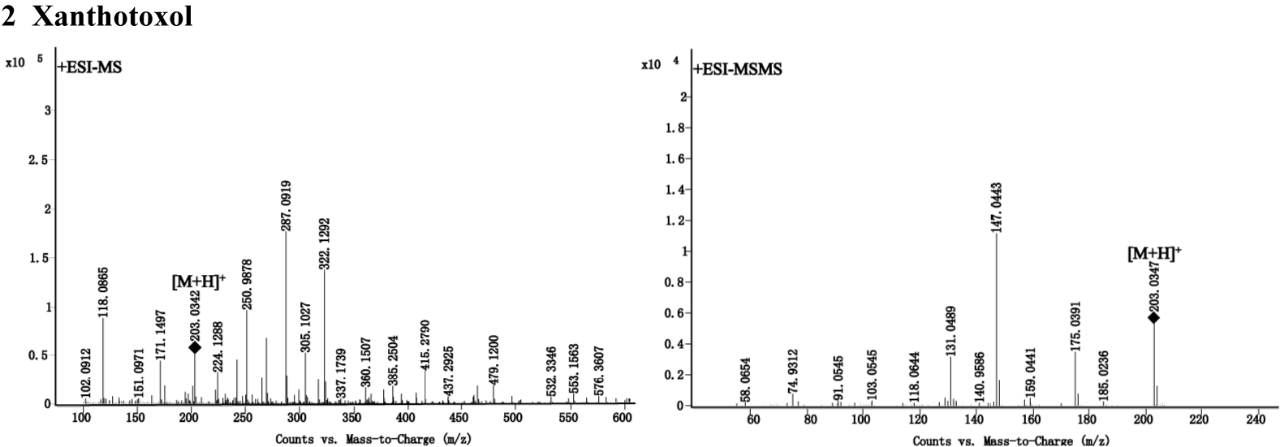


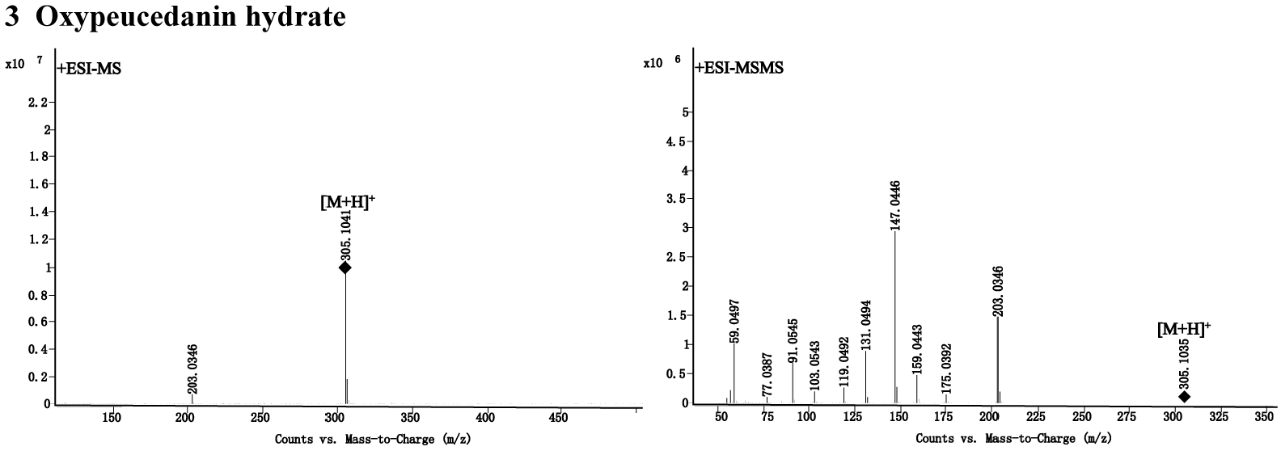


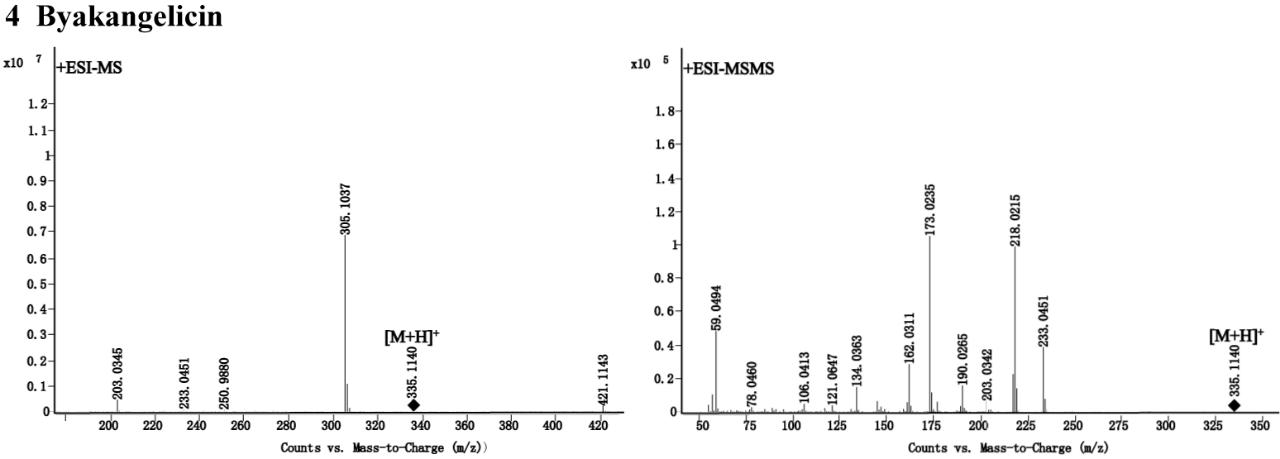


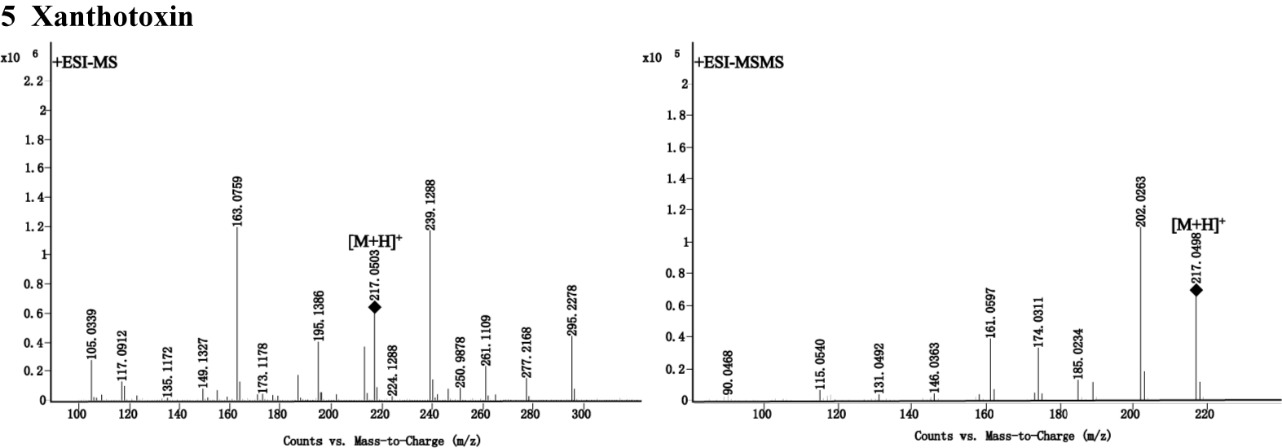


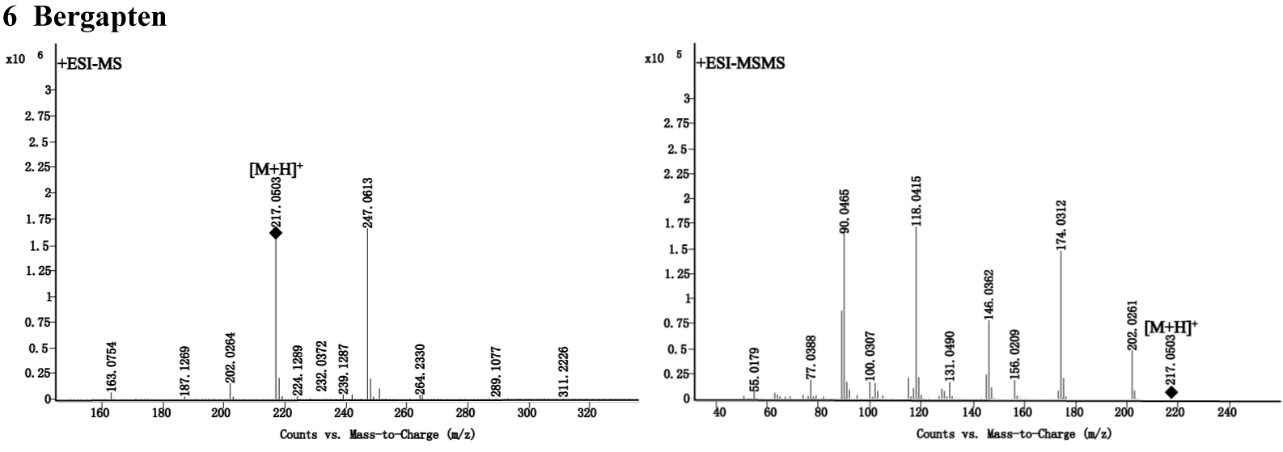


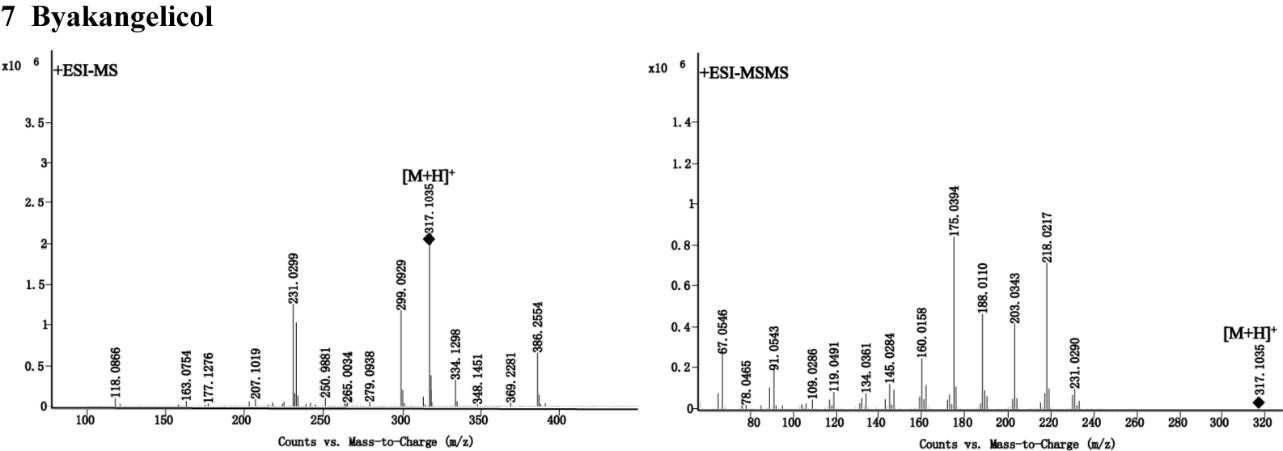


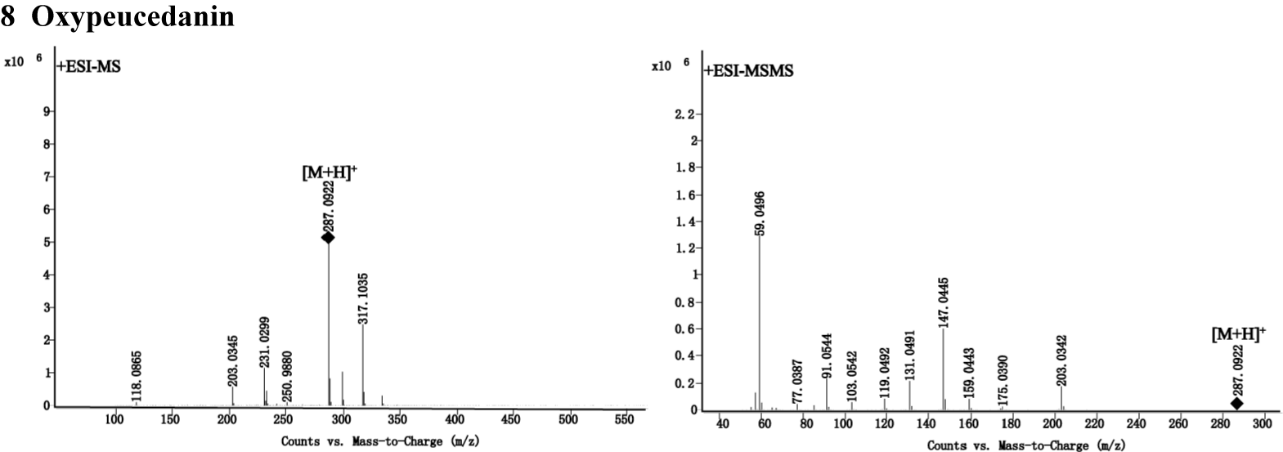


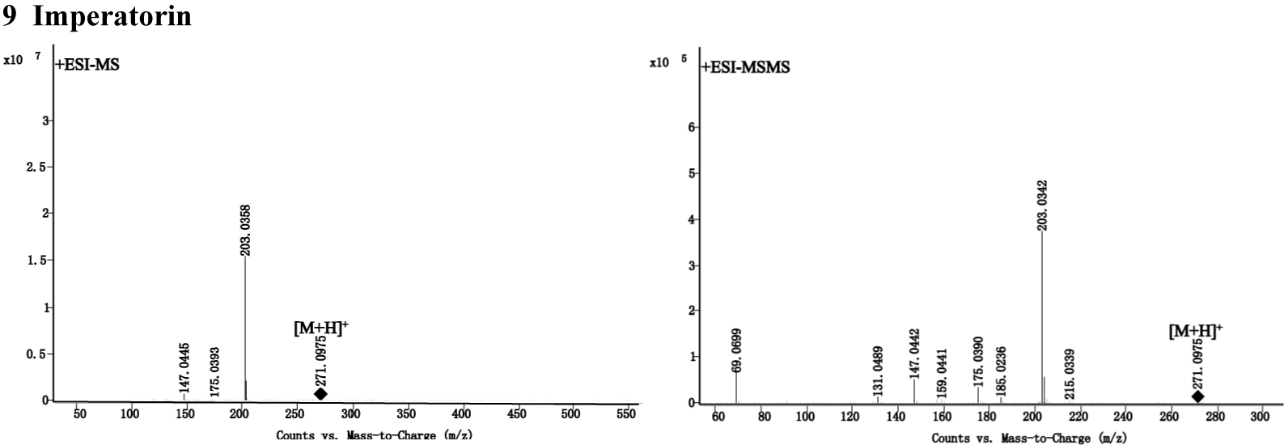


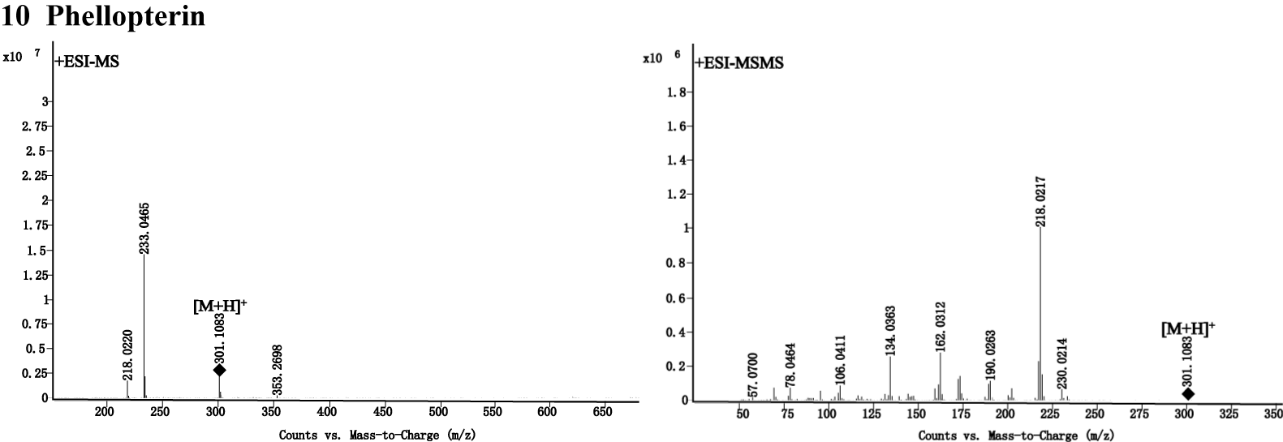


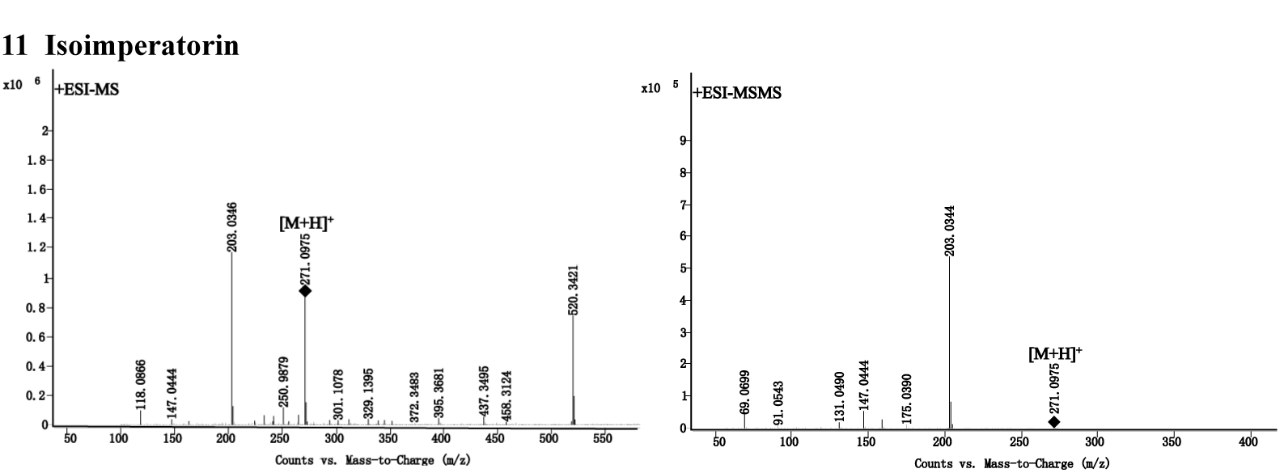


**Supplementary Figure 2** The MS and MS/MS spectrums of eleven coumarins identified in ADR.


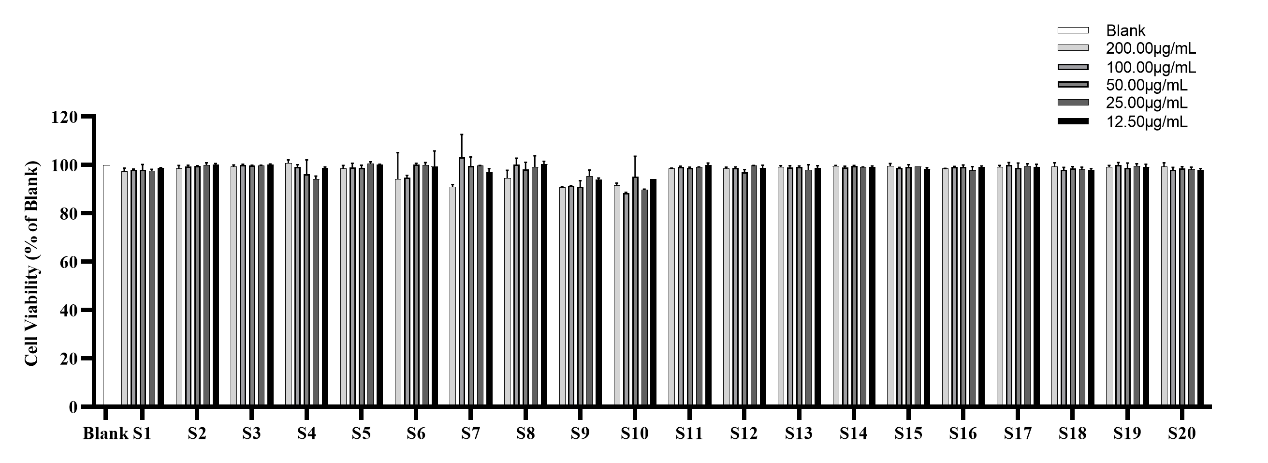


**Supplementary Figure 3** Effects of ADR samples extract (0, 12.5, 25, 50, 100, 200 μg/mL) on the viability of RAW 264.7 cells.


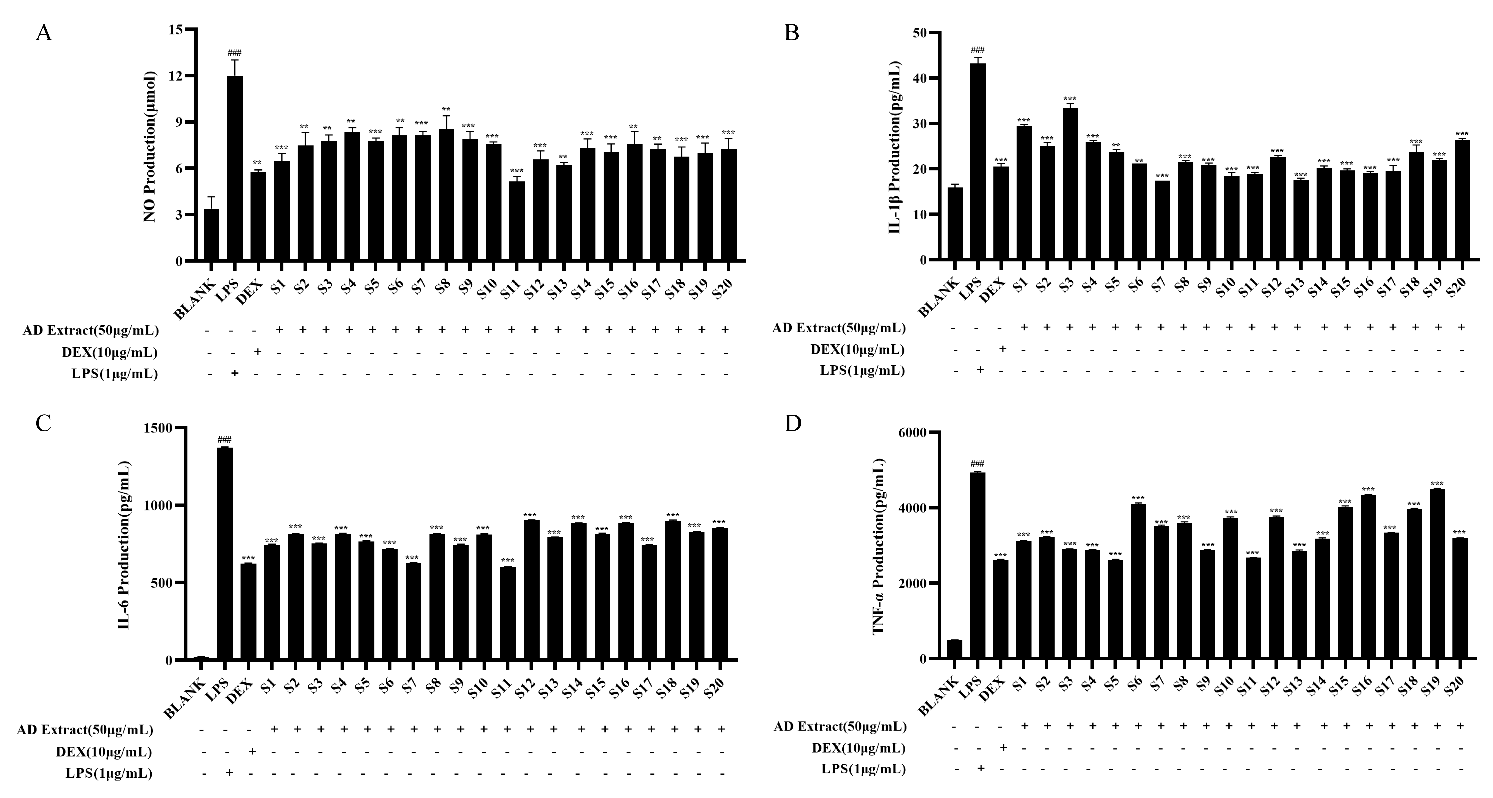
**Supplementary Figure 4** Effects of ADR samples extract (50 μg/mL) on NO (A), IL-1β (B), IL-6 (C) and TNF-α (D) production in LPS-stimulated RAW264.7 cells. ^###^ p < 0.001, ** p < 0.001, *** p < 0.001

**Supplementary Table 1** The information of twenty batches of ADR samples used in this study.

| Sample number | Origins |
| --- | --- |
| S1 | Sichuan province, China |
| S2 | Sichuan province, China |
| S3 | Sichuan province, China |
| S4 | Sichuan province, China |
| S5 | Sichuan province, China |
| S6 | Sichuan province, China |
| S7 | Sichuan province, China |
| S8 | Sichuan province, China |
| S9 | Sichuan province, China |
| S10 | Sichuan province, China |
| S11 | Anhui province, China |
| S12 | Anhui province, China |
| S13 | Anhui province, China |
| S14 | Henan province, China |
| S15 | Hubei province, China |
| S16 | Hebei. province, China |
| S17 | Hebei. province, China |
| S18 | Hebei. province, China |
| S19 | Hebei. province, China |
| S20 | Hebei. province, China |

**Supplementary Table 2** Precision, repeatability and stability of eleven common peaks.

| No. | Analyte | Precision RSD (%) | Repeatability RSD (%) | Stability RSD (%) |
| --- | --- | --- | --- | --- |
| 1 | Scopolin | 0.509 | 0.564 | 0.522 |
| 2 | Xanthotoxol | 1.112 | 0.411 | 1.145 |
| 3 | Oxypeucedanin hydrate | 1.103 | 0.375 | 0.375 |
| 4 | Byakangelicin | 1.125 | 0.403 | 0.459 |
| 5 | Xanthotoxin | 1.080 | 0.254 | 0.413 |
| 6 | Bergapten | 1.101 | 0.179 | 0.223 |
| 7 | Byakangelicol | 0.453 | 0.249 | 0.427 |
| 8 | Oxypeucedanin | 1.077 | 0.233 | 0.452 |
| 9 | Imperatorin | 1.285 | 0.170 | 0.391 |
| 10 | Phellopterin | 1.070 | 0.148 | 0.357 |
| 11 | Isoimperatorin | 1.072 | 1.971 | 1.721 |

**Supplementary Table 3**  The regression equations and linear ranges of the six coumarin compounds.

| Compounds | Regression equation | R^2^ | Linear ranges (mg/L) |
| --- | --- | --- | --- |
| Xanthotoxol | y = 9.945×10^6^x + 7731.059 | 0.9990 | 0.66-85.00 |
| Xanthotoxin | y = 2.673×10^6^x + 27132.445 | 0.9991 | 0.94-120.00 |
| Bergapten | y = 3.392×10^7^x + 16064.480 | 0.9992 | 0.47-60.00 |
| Imperatorin | y = 1.064×10^7^x + 38394.671 | 0.9992 | 1.72-220.00 |
| Phellopterin | y = 2.344×10^7^x + 47383.065 | 0.9992 | 1.99-255.00 |
| Isoimperatorin | y = 2.831×10^7^x + 56286.426 | 0.9993 | 2.03-260.00 |

**Supplementary Table 4** The peak areas of the eleven coumarins in ADR samples.

| Sample number | Scopolin | Xanthotoxol | Oxypeucedanin hydrate | Byakangelicin | Xanthotoxin | Bergapten | Byakangelicol | Oxypeucedanin | Imperatorin | Phellopterin | Isoimperatorin |
| --- | --- | --- | --- | --- | --- | --- | --- | --- | --- | --- | --- |
| S1 | 160760 | 164283 | 638464 | 410906 | 129581 | 408342 | 518685 | 1166820 | 1935563 | 873437 | 978069 |
| S2 | 154927 | 173070 | 731244 | 435172 | 153285 | 477434 | 602885 | 1379427 | 2332865 | 1010044 | 1157810 |
| S3 | 169913 | 155106 | 598151 | 349541 | 101077 | 307607 | 155106 | 1446496 | 1759327 | 826949 | 818683 |
| S4 | 623005 | 155201 | 624586 | 297477 | 107900 | 422936 | 1608992 | 4703362 | 1633072 | 1299257 | 1057290 |
| S5 | 164501 | 112049 | 542257 | 282088 | 232616 | 432540 | 469945 | 1216854 | 2476048 | 1159265 | 980693 |
| S6 | 469641 | 131444 | 275601 | 144848 | 170290 | 423370 | 1402879 | 3646490 | 1493495 | 1028023 | 1068011 |
| S7 | 587401 | 131592 | 186382 | 92579 | 117566 | 469996 | 1129501 | 3990052 | 1612462 | 1199197 | 1247479 |
| S8 | 471372 | 121112 | 235621 | 143842 | 104755 | 414426 | 1422889 | 4080501 | 1603980 | 1180834 | 1239617 |
| S9 | 424726 | 136460 | 238282 | 129168 | 159508 | 415925 | 997581 | 3137721 | 1510320 | 994185 | 1095117 |
| S10 | 601678 | 138474 | 235754 | 112328 | 129141 | 438611 | 1053246 | 3586394 | 1484921 | 1058751 | 1094648 |
| S11 | 145282 | 82193 | 528491 | 235447 | 58263 | 222016 | 308099 | 970402 | 1536425 | 729074 | 787659 |
| S12 | 155823 | 128606 | 307566 | 180826 | 82100 | 176037 | 255457 | 907796 | 1370556 | 665711 | 635679 |
| S13 | 18791 | 26812 | 454613 | 139188 | 16651 | 123071 | 162409 | 647343 | 1050726 | 546983 | 607865 |
| S14 | 49698 | 32990 | 700733 | 221096 | 35287 | 242135 | 375747 | 940254 | 1168651 | 727820 | 750896 |
| S15 | 309890 | 94064 | 430555 | 121738 | 76970 | 181206 | 211380 | 958119 | 1397599 | 719483 | 748521 |
| S16 | 131464 | 47871 | 406510 | 143559 | 31822 | 202059 | 433101 | 1041300 | 1365929 | 660728 | 731738 |
| S17 | 143520 | 159694.5 | 423399 | 167336 | 71093 | 219754 | 413347 | 1062726 | 1292308 | 681286 | 721025 |
| S18 | 127846 | 86541 | 512639 | 209124 | 31909 | 225760 | 457643 | 928495 | 1291665 | 685004 | 678546 |
| S19 | 153916 | 96707 | 417854 | 135960 | 68628 | 198423 | 329268 | 891078 | 1553377 | 726816 | 696568 |
| S20 | 128489 | 101900 | 698577 | 315264 | 28021 | 250129 | 408931 | 930631 | 1239234 | 681605 | 534804 |

**Supplementary Table 5** The cell viability of six coumarins at different concentrations on RAW264.7 macrophage cells.

| Compound | Concentrations (μM) | Cell Viability (%) | Compound | Concentrations (μM) | Cell Viability (%) |
| --- | --- | --- | --- | --- | --- |
| Xanthotoxin | 3.09 | 102.32±5.23 | Imperatorin | 2.31 | 100.55±0.35 |
|  | 6.18 | 98.93±3.04 |  | 4.62 | 97.32±2.87 |
|  | 12.37 | 108.07±2.29 |  | 9.25 | 100.00±1.38 |
|  | 24.73 | 92.71±1.29 |  | 18.50 | 93.07±3.73 |
|  | 49.46 | 97.13±2.28 |  | 37.00 | 98.84±2.36 |
|  | 98.93 | 97.62±4.22 |  | 74.00 | 96.24±3.93 |
| Xanthotoxol | 2.89 | 97.96±3.29 | Phellopterin | 2.08 | 102.88±1.37 |
|  | 5.78 | 94.69±3.35 |  | 4.16 | 99.56±5.99 |
|  | 11.56 | 99.21±0.62 |  | 8.32 | 100.42±4.37 |
|  | 23.13 | 94.89±1.62 |  | 16.65 | 96.01±0.80 |
|  | 46.26 | 98.44±0.93 |  | 33.30 | 97.64±3.18 |
|  | 92.51 | 98.98±3.33 |  | 66.60 | 101.38±0.26 |
| Bergapten | 2.89 | 99.12±1.05 | Isoimperatorin | 2.31 | 102.43±0.75 |
|  | 5.78 | 96.97±4.41 |  | 4.62 | 98.61±0.78 |
|  | 11.56 | 97.75±1.96 |  | 9.25 | 98.89±2.01 |
|  | 23.13 | 97.53±3.66 |  | 18.50 | 97.51±0.78 |
|  | 46.26 | 97.77±1.20 |  | 25.48 | 96.20±1.81 |
|  | 92.51 | 101.75±1.70 |  | 50.96 | 96.98±2.74 |
